# Fréedericksz Transitions in 6CB Based Ferronematics—Effect of Magnetic Nanoparticles Size and Concentration

**DOI:** 10.3390/ma14113096

**Published:** 2021-06-05

**Authors:** Katarína Zakutanská, Danil Petrov, Peter Kopčanský, Dorota Węgłowska, Natália Tomašovičová

**Affiliations:** 1 Institute of Experimental Physics, SAS, Watsonova 47, 040 01 Košice, Slovakia; zakutanska@saske.sk (K.Z.); kopcan@saske.sk (P.K.); 2 Physics of Phase Transitions Department, Perm State University, Bukirev St. 15, 614990 Perm, Russia; petrovda@bk.ru; 3 Institute of Chemistry, Military University of Technology, 00-908 Warsaw, Poland; dorota.weglowska@wat.edu.pl

**Keywords:** liquid crystals, magnetic nanoparticles, Fréedericksz transition

## Abstract

In this paper, results acquired from capacitance measurements performed on composites based on nematic liquid crystal 4-cyano-4′-hexylbiphenyl (6CB) and spherical iron oxide nanoparticles of various sizes are presented. Electric and magnetic Fréedericksz transitions, as well as structural transitions in combined electric and magnetic fields, were investigated. The obtained results showed the lowering of the threshold magnetic field with an increase in the volume concentration of nanoparticles. Estimations based on results obtained from measurements suggest soft anchoring between liquid crystal director and nanoparticles magnetization vector.

## 1. Introduction

Liquid crystals are unique materials with their molecules ordered to some extent while being in a liquid state. Based on the degree of ordering, several types of mesophases are known. The most studied is nematic phase in which molecules’ long axis point to one direction, but there is no positional order. The ordering of molecules results in their properties being anisotropic, due to which these remarkable materials in mesophase can be controlled by external stimuli. The application of electric or magnetic field leads to the reorientation of liquid crystal molecules. This transition is called Fréedericksz transition [1]. Control by electric field is widely used in liquid crystal displays industry due to low threshold voltage. Threshold magnetic field is too high for industrial applications because of liquid crystals diamagnetic nature. To overcome this obstacle, magnetic nanoparticles began to be added to liquid crystals. Such composite materials based on a nematic liquid crystal matrix with nanoscale magnetic admixture are known as ferronematics. Even a small amount of nanoparticles significantly influence magnetic [2,3,4], magneto-optical [5], morphological [6], dielectric [7,8,9], or electro-optical [8,9] properties. Dispersed nanoparticles enhance local short-range orientational order, order parameter, isotropic-nematic phase transition temperature, splay elastic constant [6], dielectric anisotropy [6,10,11], and dielectric permittivity [10,12], and decrease diffusion coefficient [11] and birefringence [13]. Moreover, properties relevant for display applications such as faster rising and falling time and lower threshold voltage [6,10,14] were observed. Most importantly, magnetic nanoparticles were found to be able to reduce a threshold magnetic field [2,3,4]. Lowering the threshold magnetic field, at which the response of liquid crystal molecules begins, has been the intention, since the first paper devoted to liquid crystals doped with magnetic particles was published in 1970 [15]. Since then, the composites consisting of various liquid crystals and magnetic nanoparticles have been studied experimentally [2,3,4,16,17] and theoretically [18,19,20,21,22]. Multiple experimental papers have reported [2,3,4,16] about decreasing in threshold magnetic field observed in composites of various liquid crystals and magnetic nanoparticles, but it was shown that increasing the threshold of magnetic fields can occur as well [17]. The influence of nanoparticles depends on their properties, which are related to material, size, shape, concentration, etc. Depending on the orientation of nanoparticles magnetization vector and liquid crystal director, the threshold magnetic field is reduced or increased. In the case of liquid crystal with positive diamagnetic susceptibility and nanoparticle magnetization vector parallel to the director, threshold magnetic field applied perpendicularly decreases, since magnetic nanoparticles help to rotate liquid crystal molecules via coupling between nanoparticles and liquid crystal molecules. When the initial orientation of the nanoparticles magnetization vector is perpendicular to the liquid crystal director, the threshold magnetic field is increased, because, in addition, the coupling between nanoparticles and liquid crystal molecules has to be overcome.

The present paper follows the article [23], in which we described the effect of nanoparticle size and concentration on isotropic to nematic phase transition temperature of 6CB liquid crystal doped with iron oxide nanoparticles. In the article, it was found that the isotropic to nematic phase transition temperature decreases for all the composites when compared to pure liquid crystal, but for the composites containing large amount of nanoparticles (i.e., volume concentration 10${}^{-3}$), dramatic changes occurred. A decrease in the isotropic to nematic phase transition temperature was much more pronounced and for the transition from one phase to another much wider range of temperatures was needed. Herein, electric and magnetic Fréedericksz transitions of the same composites were investigated. The aim of the paper is to elucidate the effect of nanoparticle size and concentration on Fréedericksz transition with the intention of tuning the properties of the liquid crystal matrix.

## 2. Materials and Methods

**Figure 1:**
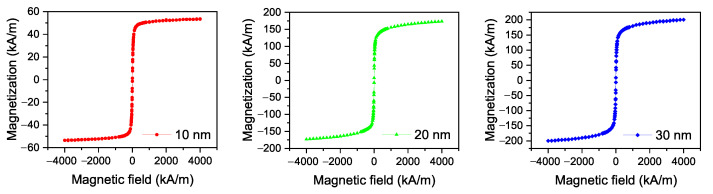
Magnetization curves of iron oxide nanoparticle powders measured at 295 K.

The iron oxide nanoparticles with diameters 10 nm, 20 nm, and 30 nm coated with oleic acid dispersed in chloroform were purchased from Ocean Nanotech. From the magnetization measurements performed on nanoparticles in powder form by SQUID magnetometer (Quantum Design MPMS 5XL), it can be seen that the nanoparticles are superparamagnetic. The magnetization curves for all three nanoparticle sizes are shown in Figure 1, from which the saturation magnetizations were determined. The saturation magnetizations of 10 nm, 20 nm, and 30 nm particles are 53 kA/m, 173 kA/m, and 200 kA/m, respectively.

The composites of thermotropic liquid crystal 4-cyano-4${}^{\prime }$-hexylbiphenyl (6CB) and iron oxide nanoparticles were prepared by the following procedure. Nanoparticles dispersed in chloroform were admixed to the liquid crystal in the isotropic phase. Subsequently, the mixture was stirred in the isotropic phase until the chloroform was evaporated. Afterward, the composite with volume concentration $10^{-3}$ remained. Diluted samples, i.e., the samples with volume concentrations $5\times 10^{-4}$ and $10^{-4}$, were acquired by admixing an additional amount of liquid crystal. Before the addition of liquid crystal, as well as before each sample preparation for measurements, the composites were sonicated to eliminate the presence of aggregates. The same procedure was repeated for nanoparticles with diameters 10 nm, 20 nm, and 30 nm leaving nine ferronematic samples—three volume concentrations for each of three nanoparticle sizes.

**Figure 2:**
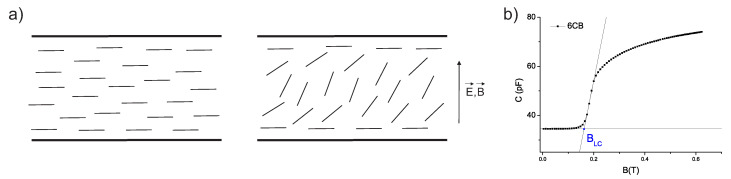
(**a**) Schematic geometry of Fréedericksz transition measurements in electric and magnetic field. (**b**) Demonstration of threshold value determination on pure 6CB liquid crystal.

**Figure 3:**
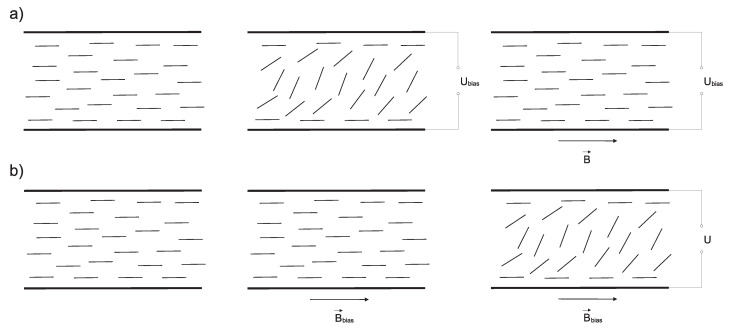
Schematic geometry for measurement of (**a**) magnetic Fréedericksz transition at applied bias voltage $U_{bias}$ and (**b**) electric Fréedericksz transition at applied bias magnetic field $B_{bias}$.

Capacitance measurements were performed on samples placed in liquid crystal cells with a 50 μm cell gap. For the measurements, the cells consisting of two glass plates with indium-tin-oxide layers serving as electrodes were filled with samples. The planar alignment of liquid crystal molecules was achieved by rubber polyimide layers. To acquire the voltage dependence of capacitance, the electric field was applied perpendicular to the cell surface. Then, similarly, the dependence of capacitance on magnetic field was obtained for magnetic field applied perpendicular to the cell surface (see Figure 2a). Finally, experiments in combined electric and magnetic fields were carried out. Electric field was applied perpendicular and magnetic field parallel to cell surface and liquid crystal molecules’ long axis’ initial orientation (see Figure 3). Firstly, the bias voltage $U_{bias}$ was higher than the threshold voltage of Fréedericksz transition, i.e., the voltage at which liquid crystal molecules start to reorient, was applied to the sample. Then, the magnetic field was gradually increased while keeping $U_{bias}$ constant. As the magnetic field was increased, the capacitance of the sample was monitored. Likewise, the dependence of capacitance on applied voltage was obtained while the constant bias magnetic field $B_{bias}$ was applied.

## 3. Results and Discussion

**Figure 4:**
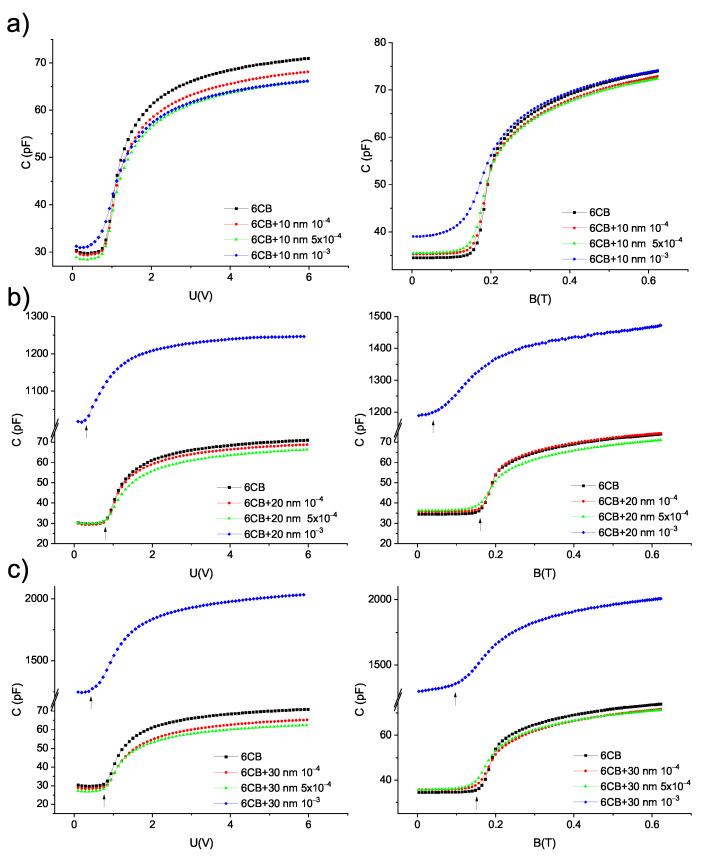
Dependencies of capacitance on electric and magnetic field for pure 6CB and composites containing (**a**) 10 nm (**b**) 20 nm (**c**) 30 nm particles.

**Figure 5:**
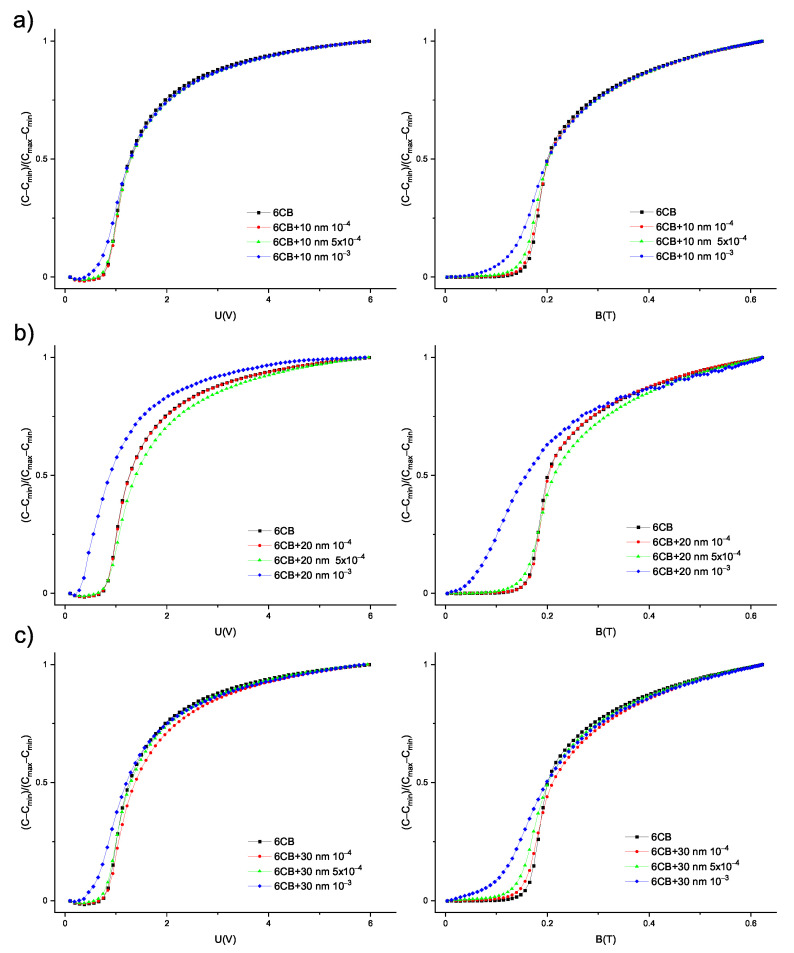
Dependencies of reduced capacitance on electric and magnetic field for pure 6CB and composites containing (**a**) 10 nm (**b**) 20 nm (**c**) 30 nm particles.

The capacitance measurements were employed to explore the Fréedericksz transition of 6CB liquid crystal and its composites. The measurements were performed at 18 °C, at which 6CB and composites were in the nematic phase. Since dielectric permittivity anisotropy and the diamagnetic susceptibility anisotropy of 6CB liquid crystal are positive, the application of external voltage and external magnetic field causes the rotation of liquid crystal molecules to the direction of the applied filed. In our experimental setting, the director was initially oriented parallel to the cell surface. The application of a large enough electric or magnetic field caused their reorientation perpendicular to the cell surface, as shown in Figure 2a. A change in orientation results in a change in capacitance. The reorientation begins when capacitance starts to increase. Threshold values are determined as the intersection of fits for two linear parts of the curves (Figure 2b) from dependencies of capacitance on voltage or magnetic field. Dependencies of capacitance on applied voltage and magnetic field are shown in Figure 4. Increasing the capacitance with increasing volume concentration of the nanoparticle can be seen. In particular, the capacitance of composites with 20 nm and 30 nm particles and volume concentration 10${}^{-3}$ is much higher (by two orders). The increase in capacitance can be caused by an increase in conductivity due to the high volume concentration of nanoparticles. A similar effect was observed for 6CB doped with Me${}_{7}$GeS${}_{5}$I (Me = Ag, Cu) nanoparticles [24]. Moreover, for these two samples, threshold voltage and magnetic field, i.e., values of voltage and magnetic field, at which the transition begins, are significantly shifted to lower values, which can be seen from the position of the arrows, indicating the beginning of Fréedericksz transition. From the curves presented in Figure 4, threshold voltage and threshold magnetic field were acquired. The values are listed in Table 1. To be able to better distinguish the shifts, the dependencies of reduced capacitance, i.e., $\left(C-C_{min}\right)/\left(C_{max}-C_{min}\right)$, where *C* is actual capacitance, $C_{min}$ is the minimum value of capacitance and $C_{max}$ is the maximum value of capacitance, on electric and magnetic field, were plotted (see Figure 5). Negligibly small changes in threshold voltage for composites compared to pure 6CB were found, with the exception of the samples with volume concentration 10${}^{-3}$, where a shift to lower values occurred. Threshold magnetic field shifted to lower values that suggest the preferred parallel orientation of magnetization vector to liquid crystal director. The curves are shifted slightly for composites with nanoparticle volume concentrations 10${}^{-4}$ and $5\times 10^{-4}$. For all the composites with volume concentration 10${}^{-3}$, the shift was much more pronounced, especially for a composite containing 20 nm particles. The shift was found to be volume concentration dependent for all three nanoparticle sizes. With higher volume concentration, the shift is more pronounced.

From the experiments, the surface density of the anchoring energy of the nematic with the particles *W* and parameter ω = $Wd/K_{1}$ can be estimated by applying Buryov and Raikher theory [25]. In the equation, *d* represents nanoparticle size and $K_{1}$ is the elastic constant of liquid crystal. The ω≲1 characterizes soft anchoring, which allows both perpendicular and parallel orientation of the magnetization vector to the director and ω≫1 characterizes rigid anchoring, which allows only parallel orientation. The well-known equation for threshold magnetic field $B_{LC}$ of nematic liquid crystal in splay geometry

(1) $$B_{LC}=\frac{\pi }{D}\sqrt{\frac{\mu_{0}K_{1}}{\chi_{a}}}$$

allows one to determine the magnetic susceptibility anisotropy $\chi_{a}$. In the equation, *D* represents the thickness of the cell gap and so the thickness of the sample, $\mu_{0}$ is the vacuum permeability, and $K_{1}$ is the elastic constant of liquid crystal. For 6CB, the elastic constant $K_{1}$ is 5.4 pN at 18 °C according to [26]. The threshold magnetic field was determined from the dependence of reduced capacitance on magnetic field at no applied voltage for pure liquid crystal 6CB. A magnetic field perpendicular to the cell surface causes the reorientation of liquid crystal molecules in its direction. The reorientation begins at the threshold magnetic field $B_{LC}$ (see Figure 2). Considering the cell thickness 50 μm, one can obtain $\chi_{a}=1.046\times 10^{-6}$. From Burylov and Raikher [25] theory, the relation between the threshold magnetic field for pure liquid crystal $B_{LC}$ and ferronematic $B_{FN}$ with the nanoparticle size *d* and volume concentration ϕ arises

(2) $$B_{FN}^{2}=B_{LC}^{2}\pm \frac{2W\phi \mu_{0}}{\chi_{a}d}$$

which enables one to estimate the value *W*, the sign of which depends on the initial orientation of the nanoparticles magnetization vector $\vec{m}$ and the director $\vec{n}$. It is negative for $\vec{m}\|\vec{n}$ and positive for $\vec{m}⊥\vec{n}$. The surface density of the anchoring energy for the ferronematic sample containing 10 nm particles, 20 nm particles and 30 nm particles was estimated to be order of $W_{10}∼10^{-8}$ Nm${}^{-1}$, $W_{20}∼10^{-7}$ Nm${}^{-1}$, and $W_{30}∼10^{-7}$ Nm${}^{-1}$. To determine type of anchoring between liquid crystal director and the vector of nanoparticles magnetization, the parameter ω was calculated. The calculated values are $\omega_{10}∼10^{-5}$, $\omega_{20}∼10^{-4}$, and $\omega_{30}∼10^{-3}$ for 10 nm, 20 nm and 30 nm particles, which respectively characterize the soft anchoring.

The weak coupling between nanoparticles magnetization vector and liquid crystal director can be confirmed by a previously published theoretical paper [27], where the equation for the dimensionless threshold field of the Fréedericksz transition $h_{c}=B_{FN}D\sqrt{\chi_{a}/\left(\mu_{0}K_{1}\right)}$ in this geometry was obtained

(3) $$\sigma =\frac{1}{2}\frac{bh_{c}\left(\pi^{2}-h_{c}^{2}\right)}{\pi^{2}-h_{c}^{2}+bh_{c}}.$$

**Figure 6:**
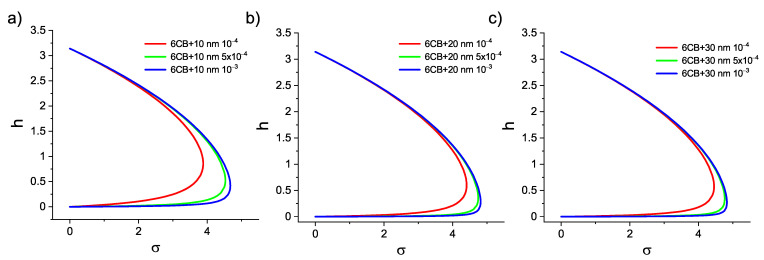
Dependencies of dimensionless field $h_{c}$ on the diemnsionless coupling energy σ for composites with (**a**) 10 nm (**b**) 20 nm (**c**) 30 nm particles.

Here, we define the dimensionless parameter $b=M_{s}\phi D\sqrt{\mu_{0}/\left(\chi_{a}K_{1}\right)}$, and the dimensionless coupling energy $\sigma =W\phi D^{2}/\left(K_{1}d\right)$. By plotting $h_{c}$ as a function of σ, the type of anchoring can be determined. The $h_{c}\left(\sigma \right)$ curves are presented in Figure 6. For all the sizes of nanoparticles, the maximum value of the dimnsionless coupling energy $\sigma_{max}$ is shifting towards higher values with the increase of the volume concentration of nanoparticles. Critical magnetic field $B_{FN}$ for all the combinations of nanoparticle sizes *d* and volume concentrations ϕ as well as calculated diemsionless parameters $h_{c}$, *b*, σ and its maximum value $\sigma_{max}$ are listed in Table 2. For the calculation, the values D=50μm, $K_{1}$ = 5.4 pN, $\chi_{a}=1.046\times 10^{-6}$, $M_{s}$ = 53,340 A/m, 173,040 A/m, and 200,340 A/m, for 10 nm, 20 nm and 30 nm particles, respectively, were used. The weak coupling is indicated by relation $\sigma <\sigma_{max}$. The definition of dimensionless coupling energy allows one to determine the surface density of the anchoring energy

(4) $$W{}_{\sigma }=\frac{\sigma \phi D^{2}}{K_{1}d}.$$

The values acquired from Equation (4) $W_{\sigma_{10}}∼10^{-8}$ Nm${}^{-1}$, $W_{\sigma_{20}}∼10^{-7}$ Nm${}^{-1}$, and $W_{\sigma_{30}}∼10^{-7}$ Nm${}^{-1}$ are in a good qualitative agreement with the values acquired by Equation (2).

**Figure 7:**
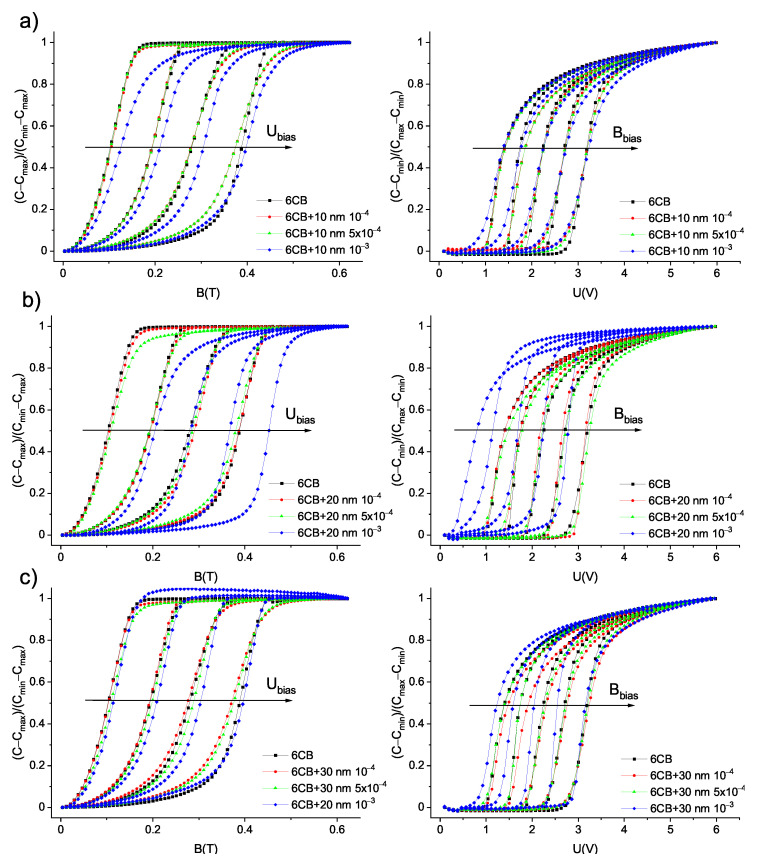
Dependencies of reduced capacitance on magnetic and electric field for various bias voltages $U_{bias}$ = 1, 1.5, 2, and 2.5 V and bias magnetic fields $B_{bias}$ = B = 0.1, 0.2, 0.3, 0.4, and 0.5 T, respectively, for pure 6CB and composites containing (**a**) 10 nm, (**b**) 20 nm (**c**), and 30 nm particles. The curves shift from the lowest bias voltage and magnetic field on the left to the highest on the right.

**Figure 8:**
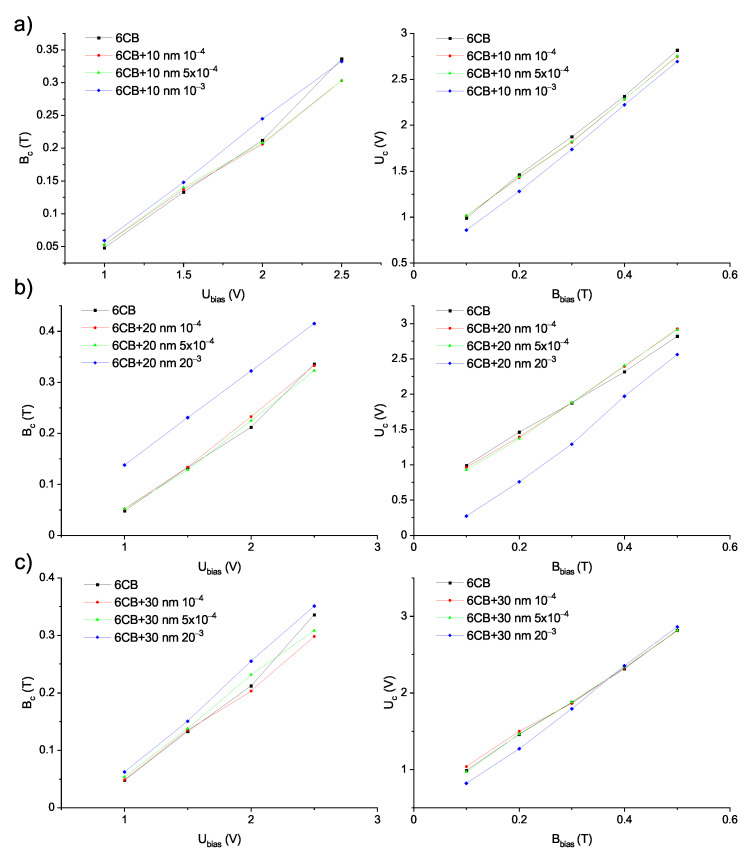
Experimental dependencies of threshold magnetic field $B_{c}$ on bias voltage $U_{bias}$ and threshold voltage $U_{c}$ on bias magnetic field $B_{bias}$, respectively, for pure 6CB and composites containing (**a**) 10 nm (**b**) 20 nm (**c**) 30 nm particles obtained from curves shown in Figure 7.

To examine how the shift in threshold voltage $U_{FN}$ affects the Fréedericksz transition in combined fields, the electric and magnetic field oriented perpendicularly to each other were applied to the samples. The measurements of capacitance dependencies on the magnetic field were performed at constant bias voltages 1, 1.5, 2, and 2.5 V, while the magnetic field was gradually increased. Since the values of bias voltage $U_{bias}$ were greater than threshold voltages $U_{LC}$ and $U_{FN}$, the bias electric field caused the reorientation of molecules, to some degree depending on the value of bias voltage and magnetic field applied perpendicular to electric field, then restoring the original alignment, as shown in Figure 3a. For the higher bias electric field, a higher magnetic threshold was observed, which was expected, since the higher bias voltage caused more pronounced rotation. Similarly, the capacitance dependencies on applied voltage were measured at bias magnetic fields 0.1, 0.2, 0.3, 0.4, and 0.5 T and voltage was gradually increased. While bias magnetic field maintains the molecules in original alignment, electric field causes the rotation of molecules perpendicularly to the cell surface (see Figure 3b). Again, threshold voltage increases with a higher bias magnetic field applied to the sample. To compare shifts in threshold magnetic fields and threshold voltages for samples with various nanoparticles volume concentrations, the dependencies of reduced capacitance on applied magnetic field and applied voltage for all the volume concentrations were plotted into one graph, along with the curve for pure 6CB (Figure 7). The corresponding dependencies of threshold magnetic field $B_{c}$ on bias voltage $U_{bias}$ and threshold voltage $U_{c}$ on bias magnetic field $B_{bias}$ are shown in Figure 8. From Figure 7 and Figure 8, we can see that the threshold magnetic field for the largest volume concentration of nanoparticles increases and threshold voltage decreases.

**Figure 9:**
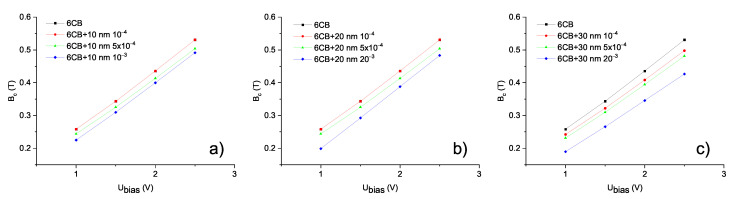
Calculated dependencies of threshold magnetic field $B_{c}$ on bias voltage $U_{bias}$ for pure 6CB and composites containing (**a**) 10 nm, (**b**) 20 nm (**c**) and 30 nm particles.

In our geometry, the addition of nanoparticles, however, should lead to lower values of the threshold magnetic field $B_{c}$ (see Figure 9), which is expressed by relation [28]

(5) $${\left(\frac{U_{bias}}{U_{F}}\right)}^{2}-{\left(\frac{B_{c}}{B_{F}}\right)}^{2}=1,$$

where $U_{bias}\geq U_{F}$. $U_{F}=U_{LC}$, $B_{F}=B_{LC}$ for liquid crystal and $U_{F}=U_{FN}$, $B_{F}=B_{FN}$ for composites. Otherwise, there is no change in orientation caused by the applied voltage that could be restored by the magnetic field. We suppose that the discrepancy between data obtained from the experiment and from the Equation (5) is caused by a significant shift in threshold voltage in the absence of the magnetic field $U_{FN}$ to lower values. Since the reorientation of molecules caused by electric field in ferronematic samples with a large concentration begins at lower values, the same bias voltage $U_{bias}$ causes a more pronounced rotation of molecules and therefore a greater magnetic field is needed to restore the initial orientation.

## 4. Conclusions

The experiments focused on electric and magnetic Fréedericksz transition in composites containing 10 nm, 20 nm, and 30 nm iron oxide nanoparticles with volume concentrations of nanoparticles $10^{-4}$, $5\times 10^{-4}$, and $10^{-3}$ showed the lowering of threshold voltage and threshold magnetic field with increasing volume concentration for all explored sizes of nanoparticles. The decrease in threshold values, which is required from an application point of view, was the most significant for the composites, with the highest nanoparticle volume concentration. Although, in these composites, the shift to lower values is the most pronounced, a large number of nanoparticles in composites causes the disruption of other properties, such as a drastic change in isotropic to nematic phase transition temperature, as we showed in our previous article [23], where moreover, sharp isotropic to nematic phase transition in the pure liquid crystal was replaced by transition stretched over a wide range of temperatures in the composites.

Based on the experiments conducted in the electric and magnetic field, the type of anchoring between liquid crystal and nanoparticles surface was found to be soft. Soft anchoring allows both the parallel and perpendicular orientation of nanoparticles magnetization vector with respect to the director. Lowering the threshold magnetic field in the absence of voltage, however, indicates parallel orientation.

## Figures and Tables

**Table 1:** Threshold voltage $U_{FN}$ and threshold magnetic field $B_{FN}$ for composites acquired from data presented in Figure 4. The threshold voltage of pure 6CB is $U_{LC}$ = 0.79 V and threshold magnetic field $B_{LC}$ = 0.16 T.

	$U_{\mathrm{FN}}$ (V)			$B_{\mathrm{FN}}$ (T)		
	10 nm	20 nm	30 nm	10 nm	20 nm	30 nm
$10^{-4}$	0.79	0.79	0.79	0.16	0.16	0.15
$5\times 10^{-4}$	0.78	0.78	0.76	0.15	0.15	0.14
$10^{-3}$	0.63	0.26	0.54	0.12	0.05	0.09

**Table 2:** Table of parameters—volume concentration ϕ, threshold magnetic field $B_{FN}$, dimensionless threshold field $h_{c}$, dimensionless parameter *b*, and the dimensionless coupling energy σ and its maximum value $\sigma_{max}$.

*d* (nm)	ϕ	$B_{\mathrm{FN}}$ (T)	$h_{c}$	*b*	σ	$\sigma_{\max}$
	$10^{-4}$	0.16	3.14	126	0.022	3.88
10 nm	$5\times 10^{-4}$	0.15	2.94	629	0.60	4.53
	$10^{-3}$	0.12	2.36	1258	2.16	4.67
	$10^{-4}$	0.16	3.14	408	0.022	4.40
20 nm	$5\times 10^{-4}$	0.15	2.94	2040	0.60	4.74
	$10^{-3}$	0.05	0.98	4080	4.44	4.81
	$10^{-4}$	0.15	2.94	472	0.60	4.45
30 nm	$5\times 10^{-4}$	0.14	2.75	2362	1.16	4.76
	$10^{-3}$	0.09	1.77	4725	3.37	4.82

## Data Availability

The data presented in this study are available within this article. Further inquiries may be directed to the authors.

## Abbreviations

*b*	dimensionless parameter	(-)
*B*	mgnetic field	(T)
*C*	capacitance	(pF)
*d*	diameter of nanoparticle	(nm)
*D*	cell gap	(μm)
$h_{c}$	dimensionless threshold field	(-)
$K_{1}$	splay elastic constant	(pN)
$\vec{m}$	unit vector of the magnetization
$M_{s}$	saturation magnetization	(A/m)
$\vec{n}$	director
*U*	voltage	(V)
*W*	anchoring energy	(N/m)
$\mu_{0}$	vacuum permeability	(H/m)
σ	dimensionless energy	(-)
ϕ	volume concentration	(-)
$\chi_{a}$	magnetic susceptibility anisotropy	(-)
**Subscripts**
c	threshold value for measurements in combined
	electric and magnetic field
F	threshold value for measurements in electric and
	magnetic field separately
FN	ferronematic
LC	liquid crystal
max	maximum value
min	minimum value
**Abbreviations**
6CB	4-cyano-4${}^{\prime }$-hexylbiphenyl
SQUID	superconducting quantum interference device
